# Supplementary figures and images for: A novel perceptual two layer image fusion using deep learning for imbalanced COVID-19 dataset
Source: PeerJ Comput Sci. 2021 Feb 10;7:e364. doi: 10.7717/peerj-cs.364 (PMC7959632; doi:10.7717/peerj-cs.364)

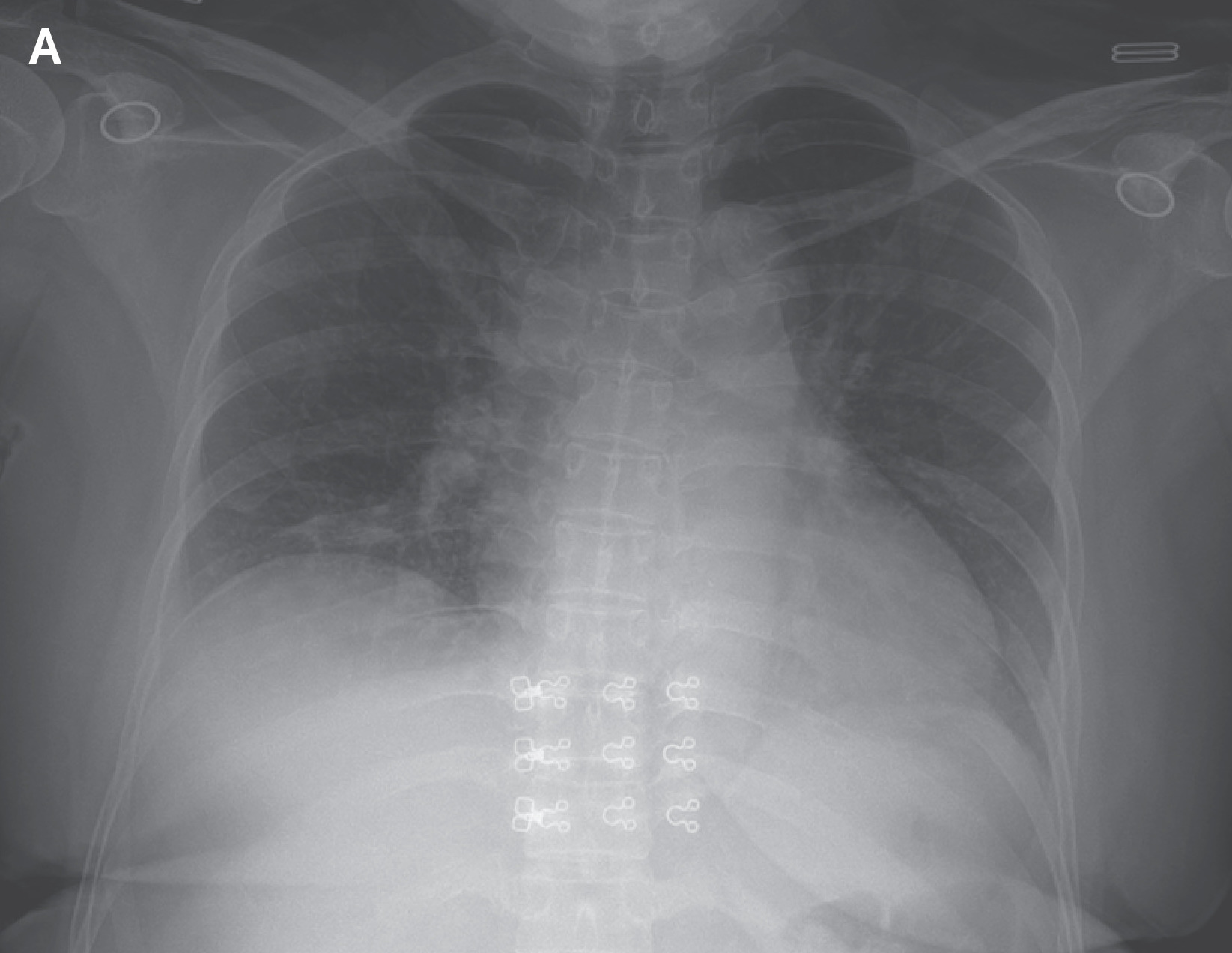

Supplement: Supplemental Information 1 [file peerj-cs-07-364-s001.zip › 4F/A.jpeg]

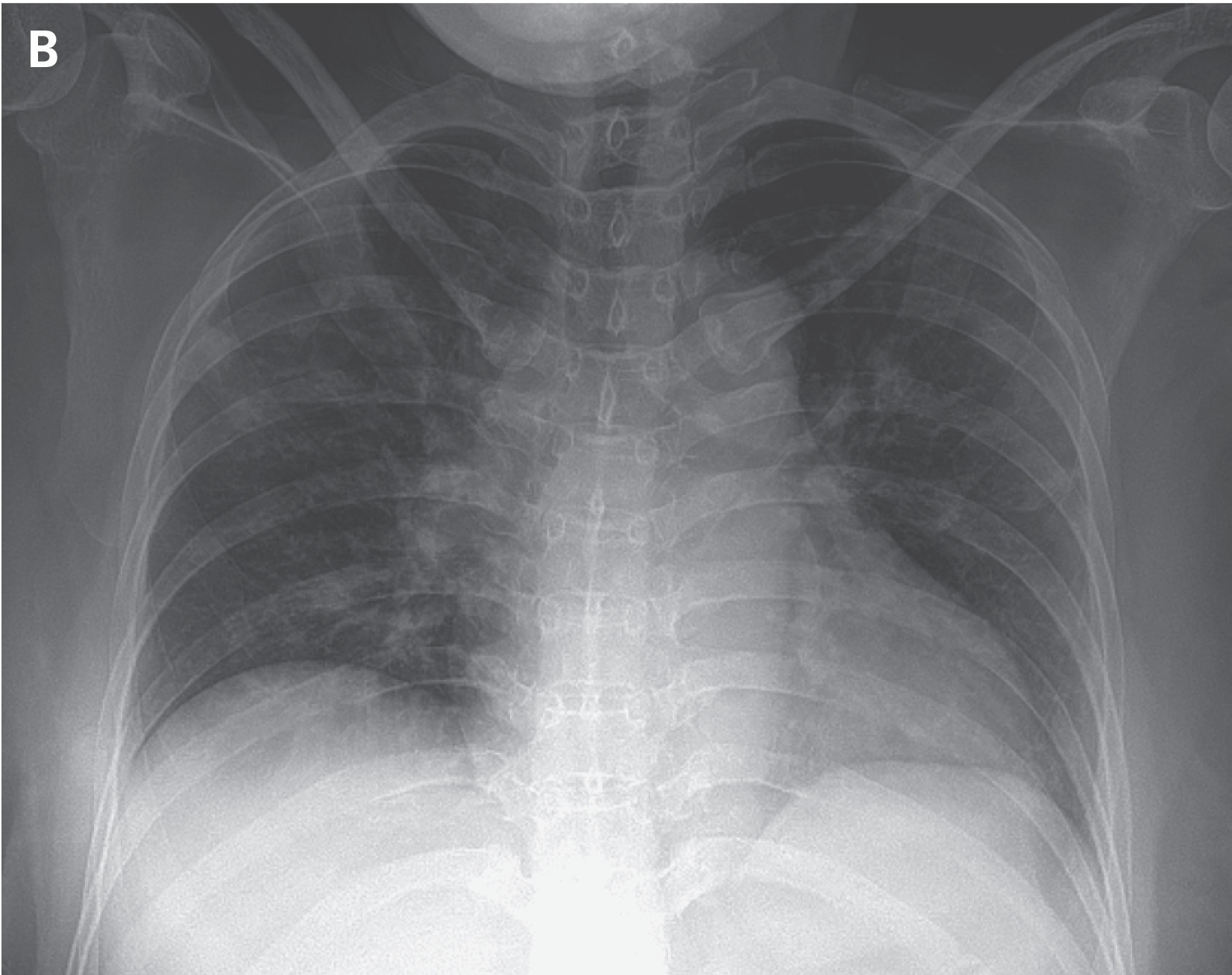

Supplement: Supplemental Information 1 [file peerj-cs-07-364-s001.zip › 4F/B.jpeg]

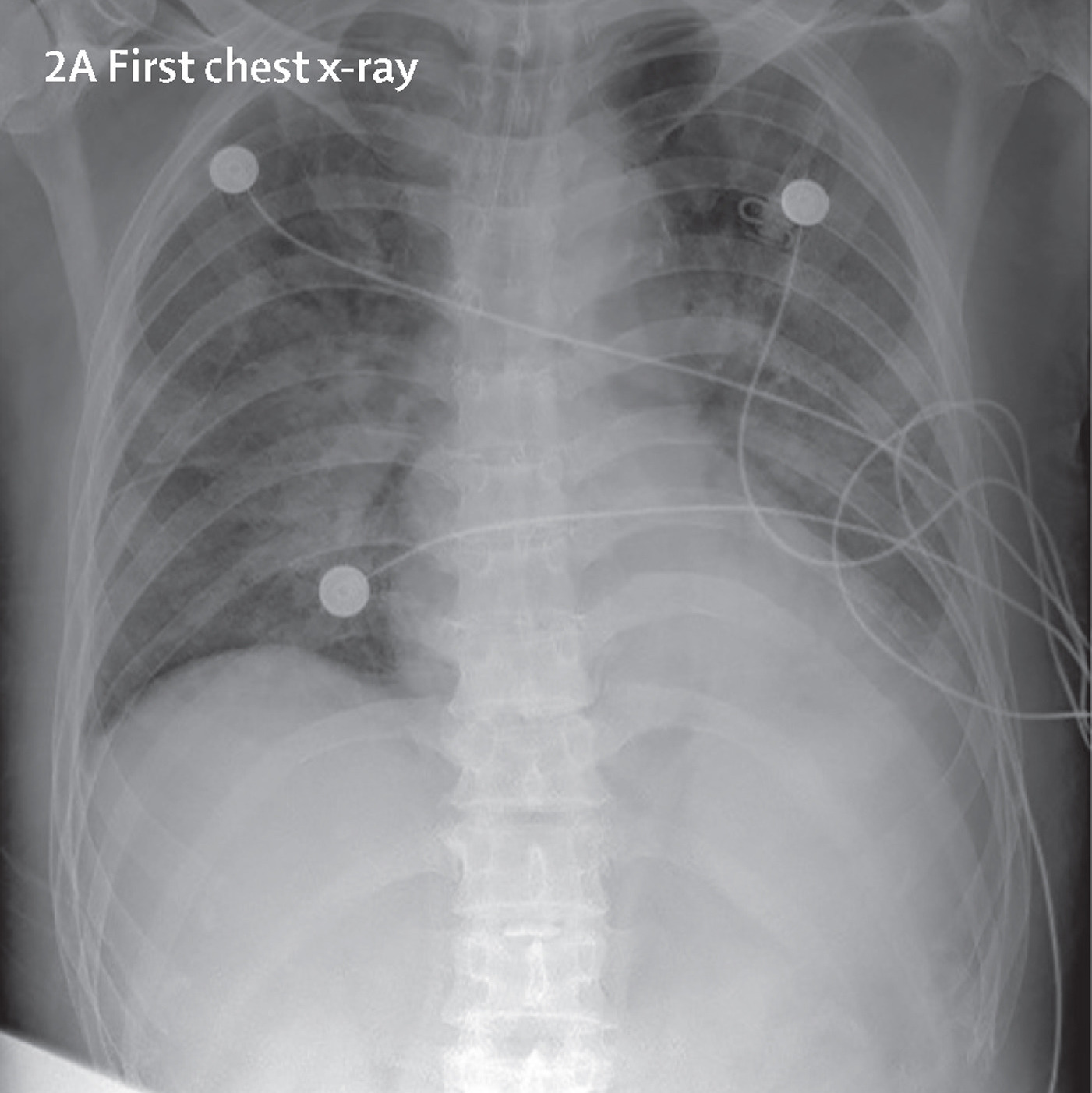

Supplement: Supplemental Information 1 [file peerj-cs-07-364-s001.zip › 6/A.jpg]

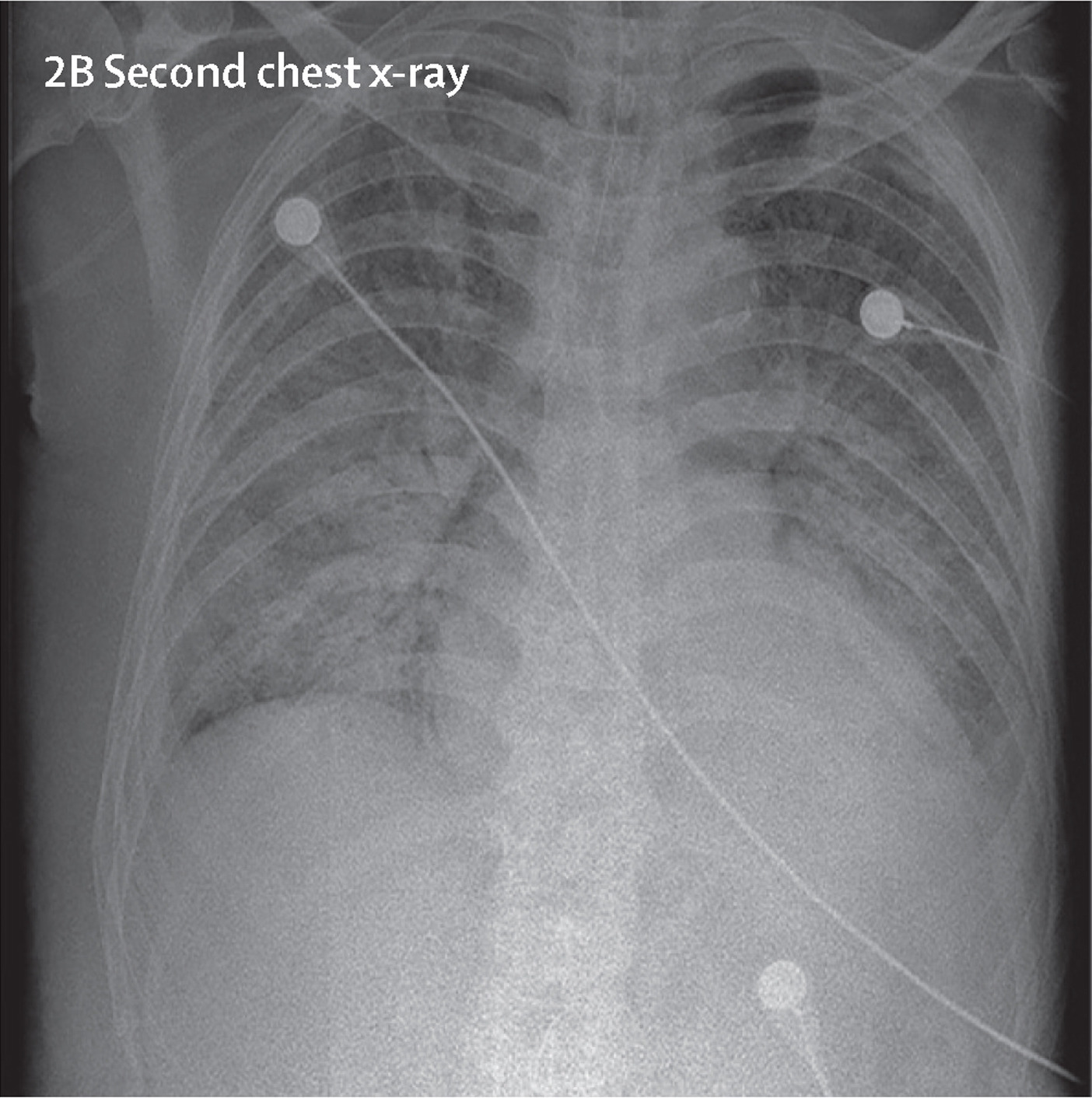

Supplement: Supplemental Information 1 [file peerj-cs-07-364-s001.zip › 6/B.jpg]

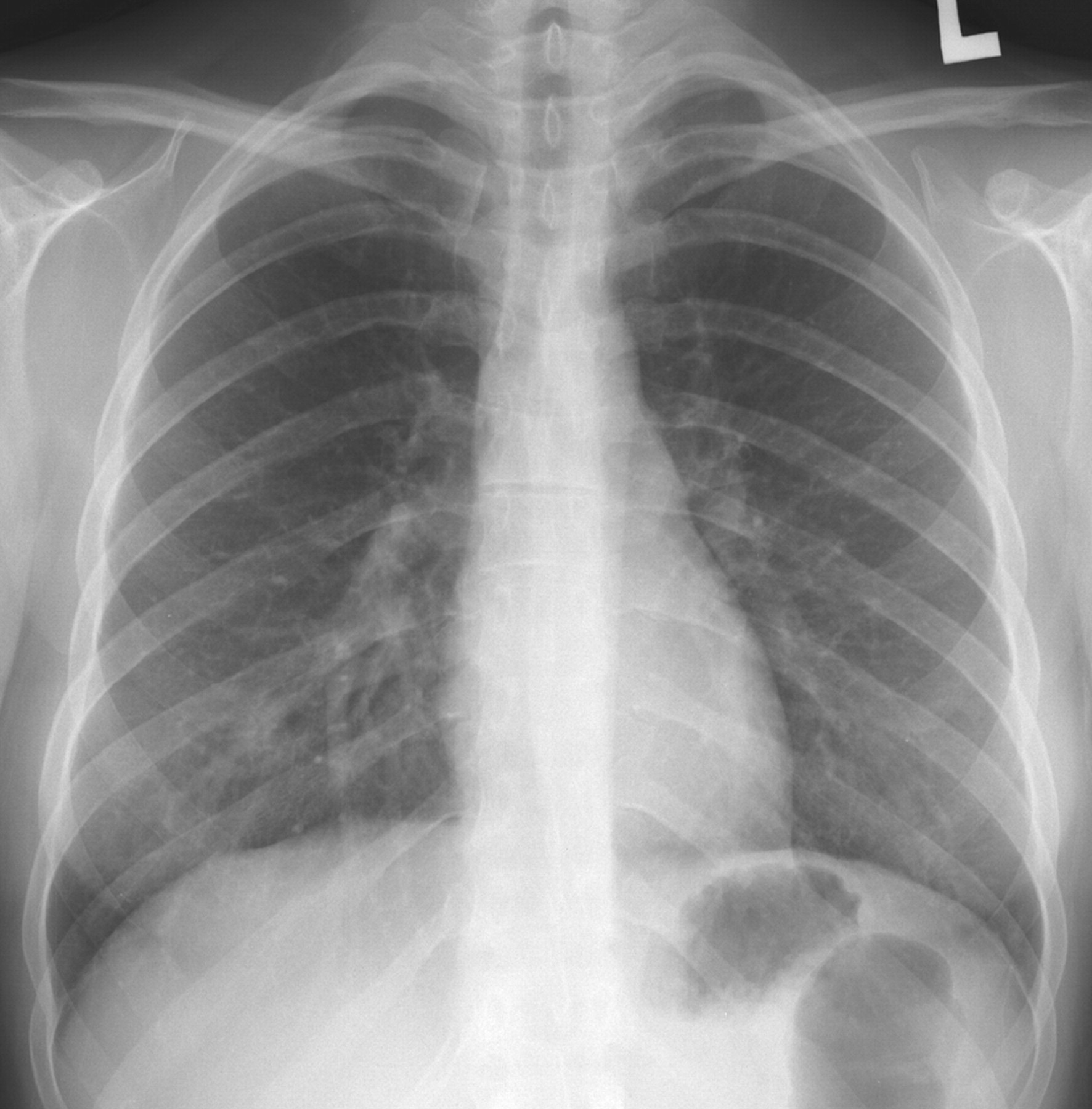

Supplement: Supplemental Information 1 [file peerj-cs-07-364-s001.zip › 7/A.jpeg]

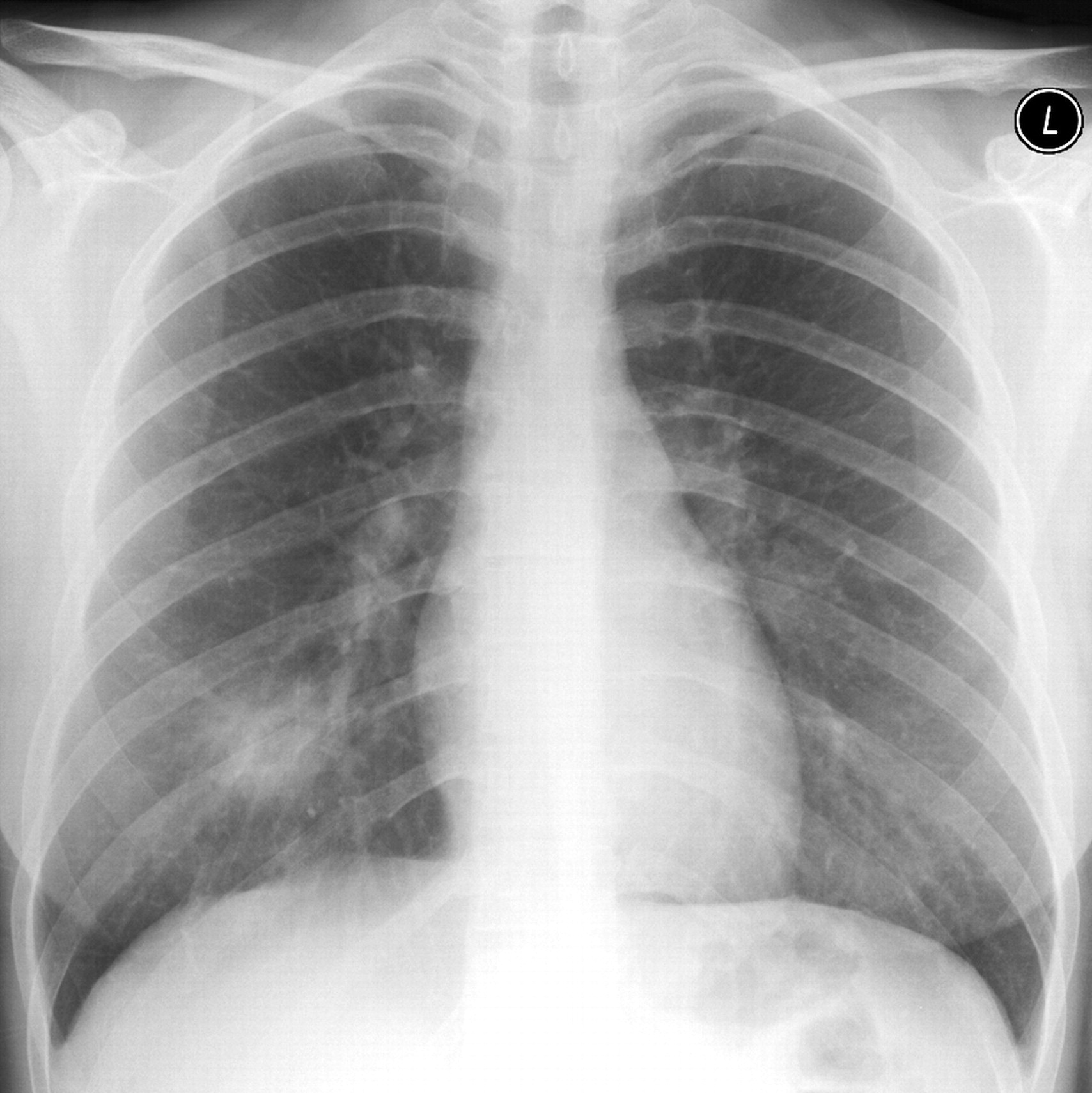

Supplement: Supplemental Information 1 [file peerj-cs-07-364-s001.zip › 7/B.jpeg]

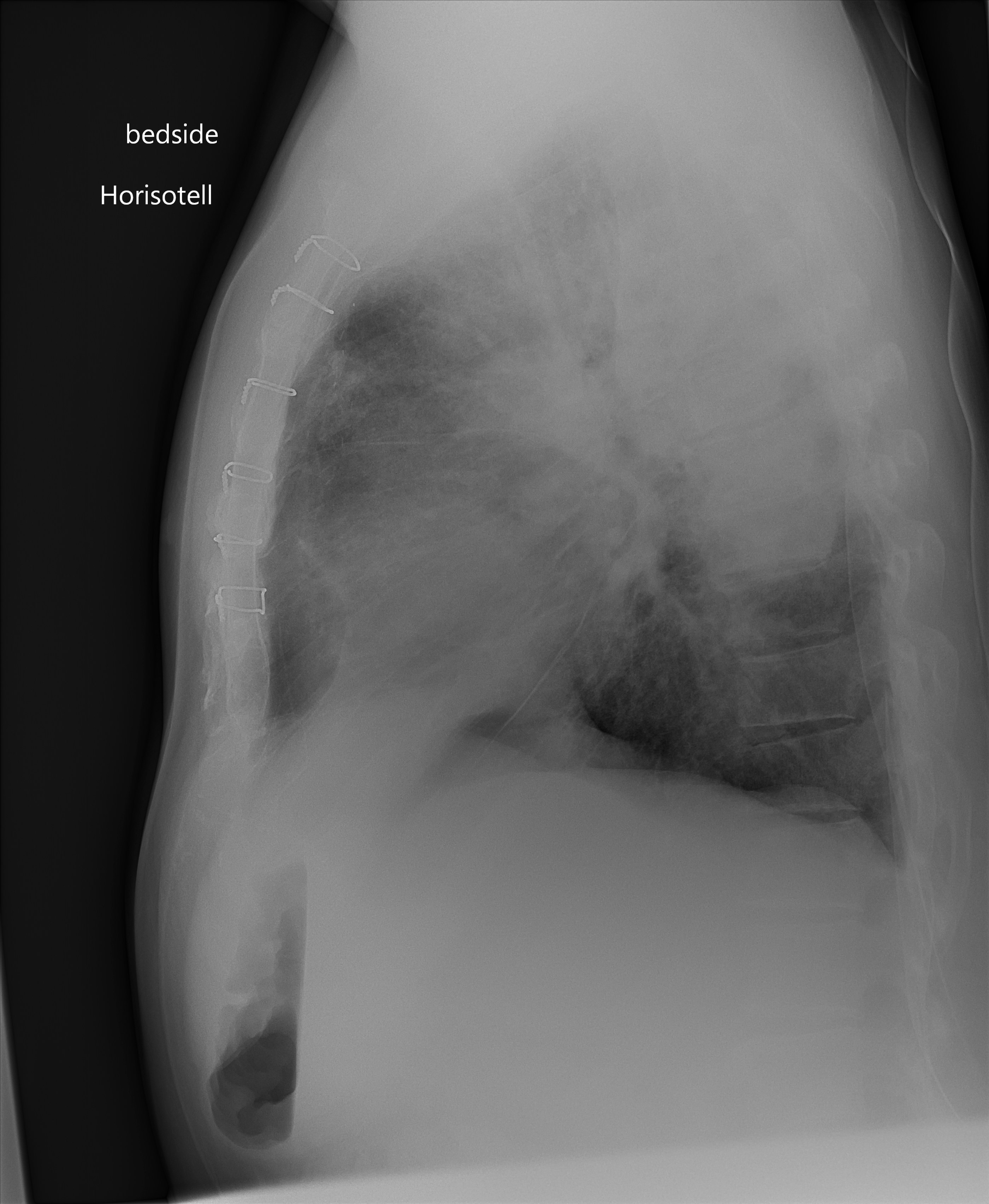

Supplement: Supplemental Information 1 [file peerj-cs-07-364-s001.zip › 20/A.jpg]

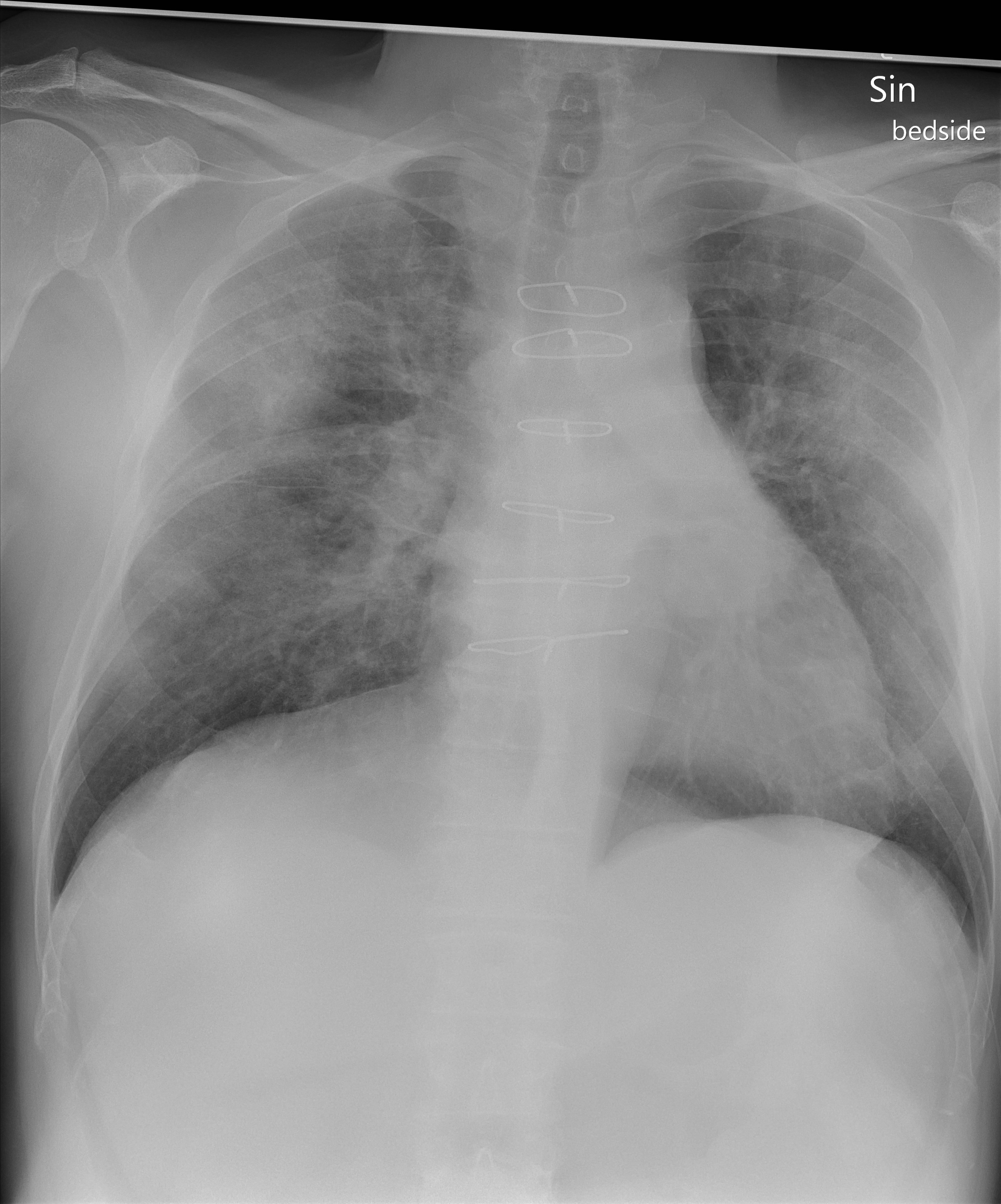

Supplement: Supplemental Information 1 [file peerj-cs-07-364-s001.zip › 20/B.jpg]

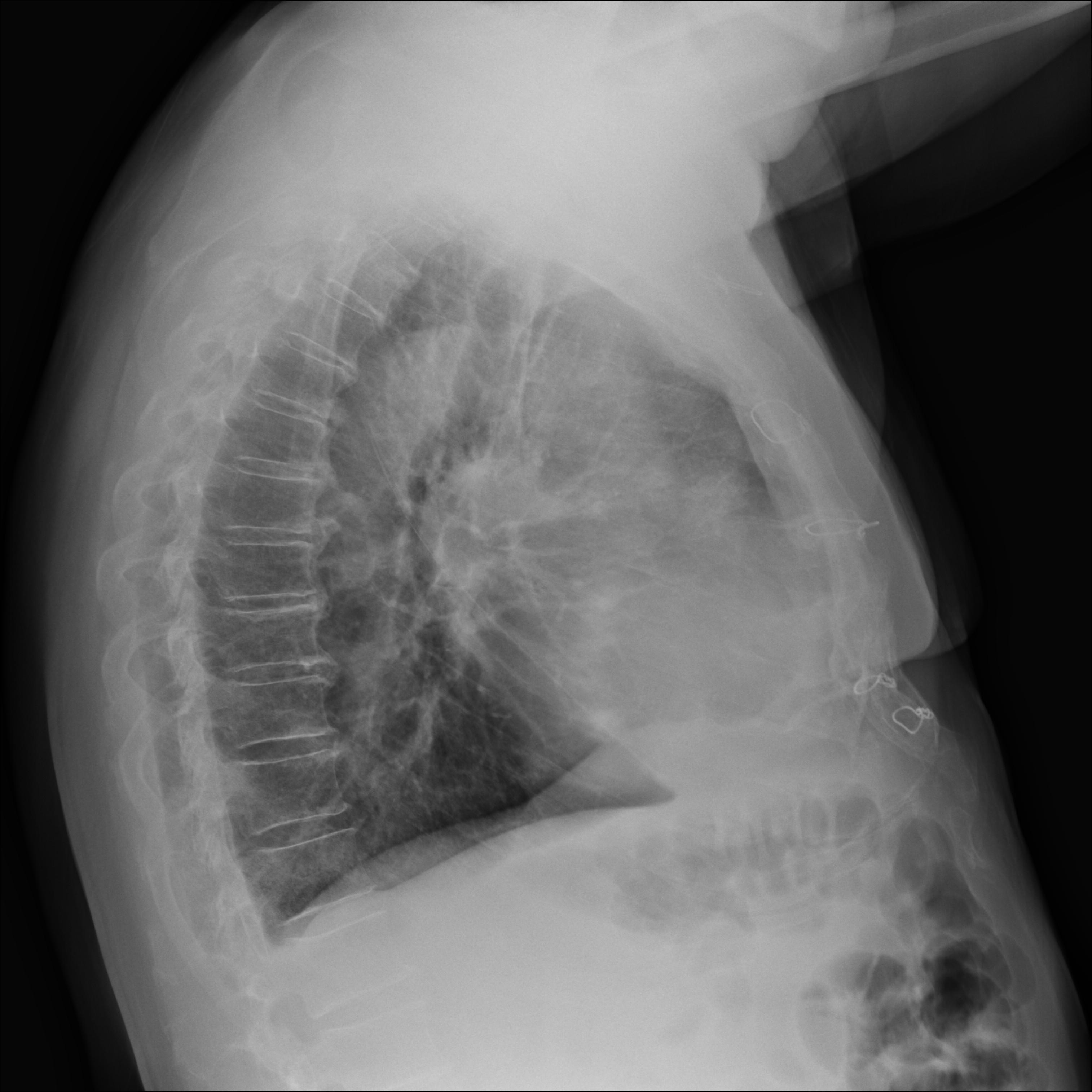

Supplement: Supplemental Information 1 [file peerj-cs-07-364-s001.zip › 22/A.jpg]

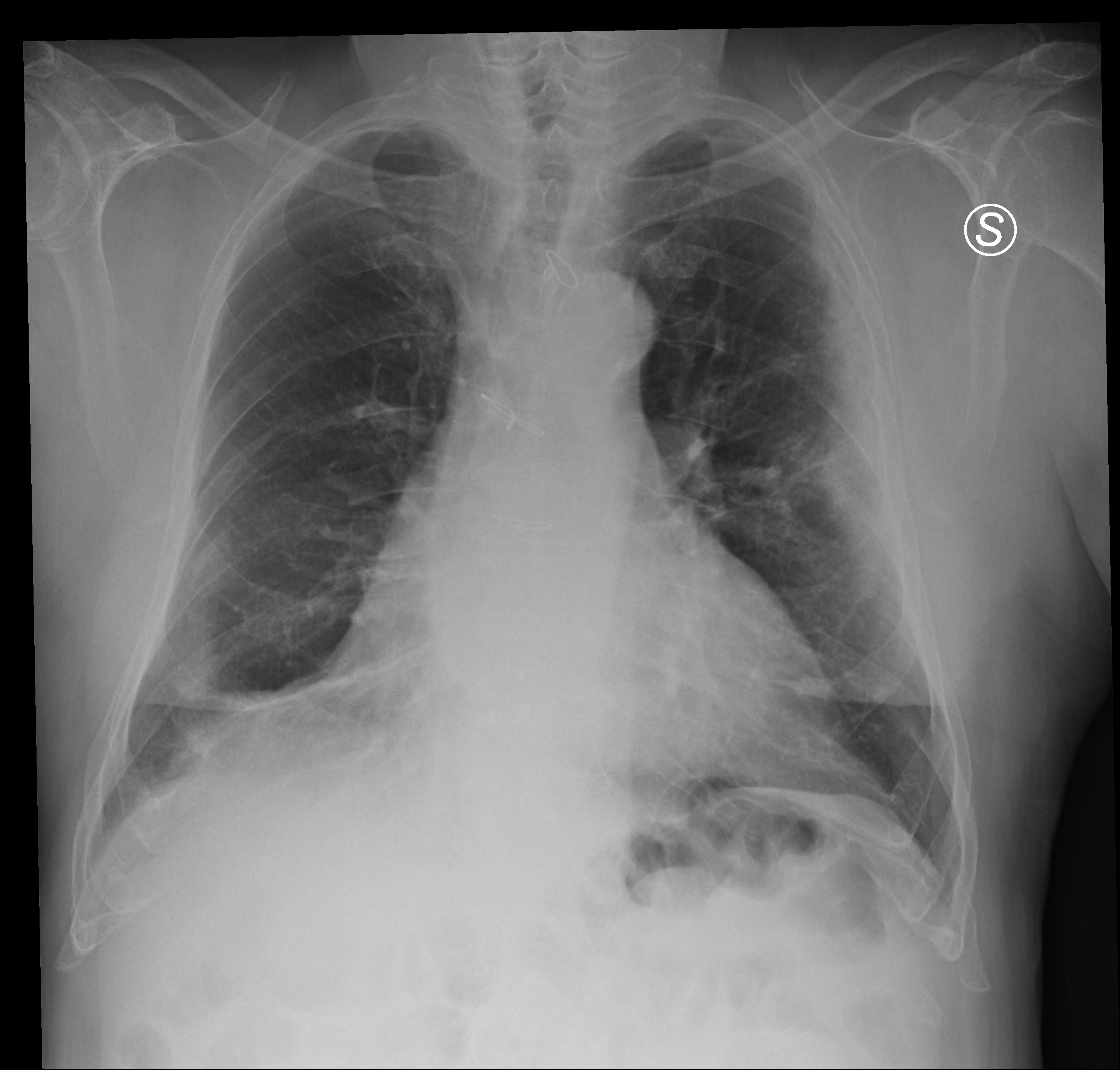

Supplement: Supplemental Information 1 [file peerj-cs-07-364-s001.zip › 22/B.jpg]

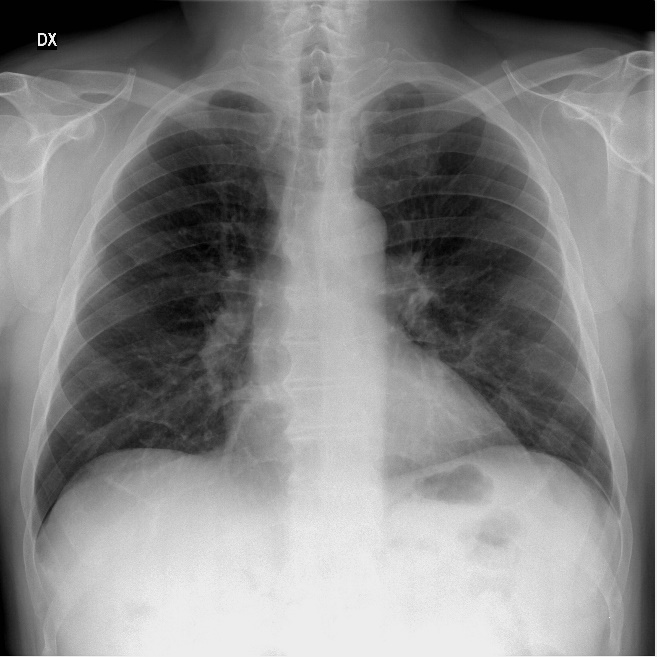

Supplement: Supplemental Information 1 [file peerj-cs-07-364-s001.zip › 36 M/A.jpeg]

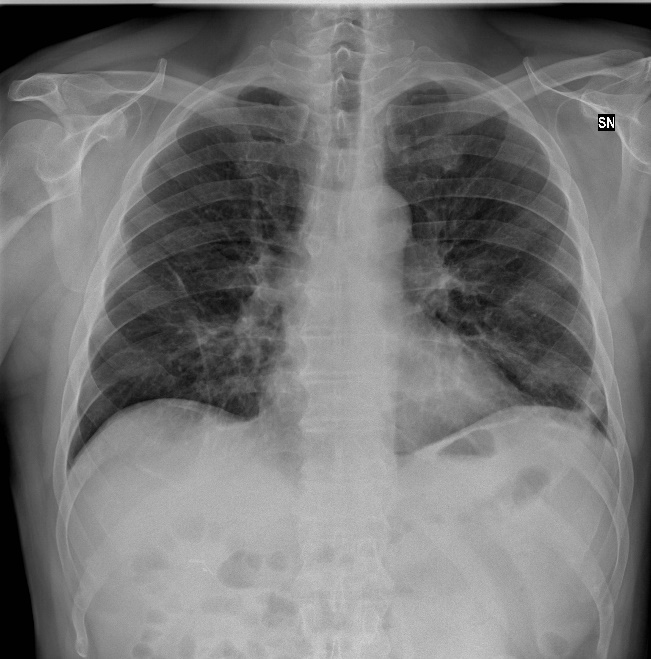

Supplement: Supplemental Information 1 [file peerj-cs-07-364-s001.zip › 36 M/B.jpeg]

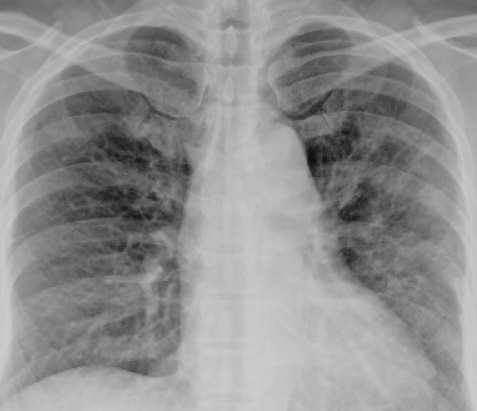

Supplement: Supplemental Information 1 [file peerj-cs-07-364-s001.zip › 59/A.jpg]

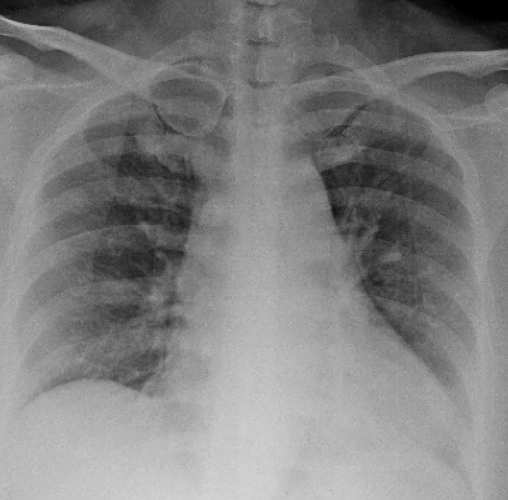

Supplement: Supplemental Information 1 [file peerj-cs-07-364-s001.zip › 59/B.jpg]
